# Relationship Between the Systemic Coagulation–Inflammation Index and Radial Artery Occlusion After Transradial Coronary Angiography

**DOI:** 10.14740/cr2275

**Published:** 2026-08-31

**Authors:** Onur Altinkaya, Rauf Macit

**Affiliations:** a Department of Cardiology, Erzurum City Hospital, Erzurum, Türkiye; b Department of Cardiology, Kagizman State Hospital, Kars, Türkiye

**Keywords:** Radial artery occlusion, Transradial access, Systemic Coagulation–Inflammation Index, Coronary angiography, Inflammation

## Abstract

**Background:**

Radial artery occlusion (RAO) is the most common vascular complication of transradial coronary procedures. The Systemic Coagulation–Inflammation Index (SCII), which integrates platelet count, fibrinogen, and white blood cell count, jointly reflects coagulation activation and systemic inflammation, two central mechanisms in RAO. We aimed to evaluate the association between SCII and RAO and its predictive value.

**Methods:**

This single-center, retrospective cohort study included 2,055 patients who underwent elective transradial coronary angiography or percutaneous coronary intervention between January 2021 and December 2025. Radial artery patency was assessed by Doppler ultrasonography before discharge, and RAO was defined as the absence of antegrade flow. SCII was calculated as (platelet count × fibrinogen)/(white blood cell count × 100). Predictors of RAO were identified using logistic regression, and the discriminative performance of SCII was assessed by receiver operating characteristic (ROC) analysis.

**Results:**

RAO occurred in 96 patients (4.7%). Patients who developed RAO were younger (55.4 ± 11.4 vs. 60.5 ± 12.1 years, P < 0.001), more frequently diabetic (11.5% vs. 5.0%, P = 0.008), and had higher SCII values (120.7 ± 36.4 vs. 93.1 ± 41.5, P < 0.001). In multivariable analysis, age (odds ratio (OR): 0.966; 95% confidence interval (CI): 0.949–0.983; P < 0.001), diabetes mellitus (OR: 2.342; 95% CI: 1.203–4.560; P = 0.012), and SCII (per 10-unit increase; OR: 1.174; 95% CI: 1.120–1.228; P < 0.001) were independent predictors of RAO, whereas sex, percutaneous coronary intervention, C-reactive protein, and creatinine lost significance. SCII showed a moderate discriminative ability (AUC: 0.730; 95% CI: 0.686–0.774; P < 0.001) with a greater AUC than other inflammatory indices. A cut-off of 101.2 yielded 70.4% sensitivity and 57.7% specificity.

**Conclusions:**

SCII is an independent predictor of RAO and shows moderate discriminative ability in patients undergoing transradial coronary procedures. As a marker readily derived from routine laboratory parameters, SCII may serve as a useful adjunct to existing clinical and procedural risk factors for the early identification of patients at increased risk of RAO. Multicenter, prospective studies are warranted to confirm these findings.

## Introduction

Transradial access is currently the preferred vascular approach for coronary angiography (CAG) and percutaneous coronary intervention (PCI) because it is associated with lower rates of bleeding and vascular complications, greater patient comfort, and improved clinical outcomes in selected patient populations [1]. Despite these advantages, radial artery occlusion (RAO) remains the most frequent access-site complication following transradial procedures [2]. The reported incidence of RAO ranges from approximately 1% to 10%, depending on the timing of assessment and the diagnostic method used [3]. Although RAO is often clinically silent owing to the dual arterial blood supply of the hand, its occurrence may prevent future transradial procedures, limit the use of the radial artery as a conduit for coronary artery bypass grafting, and complicate the creation of arteriovenous fistulas in patients requiring hemodialysis [4]. Consequently, identifying patients at increased risk of RAO before the procedure remains an important clinical challenge.

The development of RAO is a multifactorial process involving endothelial injury, vascular spasm, reduced arterial flow, and subsequent thrombus formation [3]. Increasing evidence suggests that inflammation and coagulation are closely interconnected and play pivotal roles in both thrombus formation and vascular occlusion [5]. For this reason, several inflammatory and immune-related biomarkers have been evaluated as potential predictors of cardiovascular events and vascular complications [6]. However, most currently available markers primarily reflect inflammatory activity and provide limited information regarding coagulation status, despite the fact that both processes contribute substantially to the pathogenesis of RAO.

The Systemic Coagulation–Inflammation Index (SCII) is a relatively novel biomarker that integrates platelet count, fibrinogen concentration, and white blood cell (WBC) count into a single index, thereby reflecting the interaction between coagulation activation and systemic inflammation. Recent studies have demonstrated the prognostic value of SCII in a variety of clinical settings, including in-stent restenosis after drug-eluting stent implantation [7], septic embolism in patients with infective endocarditis [8], bleeding complications in acute coronary syndrome [9], mortality in acute type A aortic dissection [10], and early arteriovenous fistula failure in patients undergoing hemodialysis [11]. Given the central role of both inflammation and thrombosis in the development of RAO, SCII may provide valuable information for identifying patients at increased risk of this complication following transradial procedures. Nevertheless, the relationship between SCII and RAO has not yet been investigated.

Accordingly, the present study was designed to evaluate the association between SCII and RAO in patients undergoing transradial CAG or PCI and to determine the predictive value of SCII for the development of RAO.

## Materials and Methods

### Study population and design

This single-center, retrospective observational cohort study included consecutive patients who underwent transradial CAG or PCI for elective indications at our institution between January 2021 and December 2025. A total of 2,055 patients were enrolled. Demographic characteristics, clinical data, laboratory parameters, and procedural details were obtained retrospectively from the hospital electronic records. The primary endpoint was the development of RAO after the procedure.

Patients presenting with acute coronary syndrome—including ST-segment elevation myocardial infarction, non–ST-segment elevation myocardial infarction, or unstable angina pectoris—were excluded. Additional exclusion criteria were active infection, chronic inflammatory disease, hematological disorders, active malignancy, advanced hepatic failure, use of immunosuppressive therapy, and missing clinical or laboratory data.

The study protocol was approved by the local ethics committee (Approval No: 2025/06-175, Date: 11.06.2025). This study was conducted in compliance with the ethical standards of the responsible institution on human subjects as well as with the Declaration of Helsinki.

### Transradial procedure and hemostasis protocol

Transradial catheterization was performed by experienced interventional cardiologists using the Seldinger technique [12]. After local anesthesia with a subcutaneous injection of 2% lidocaine at the puncture site, a 6-Fr transradial sheath (Radial Introducer Set, Ares Medikal, Istanbul, Türkiye) was inserted through the radial artery via conventional access. Following sheath placement, intra-arterial unfractionated heparin (UFH) was administered—5,000 IU for diagnostic CAG and 100 IU/kg for PCI. Intra-arterial nitroglycerin (100 µg) was also given through the sheath to prevent radial artery spasm.

After the procedure, hemostasis was achieved with a transradial compression band. A patent hemostasis strategy was applied in all patients: radial artery patency was confirmed by pulse oximetry during transient compression of the ipsilateral ulnar artery, and whenever the plethysmographic signal diminished, the compression pressure was gradually reduced until antegrade flow was restored. The band was deflated stepwise by 2–3 mL every 15 min and removed after approximately 2 h. Patients were closely monitored for access-site bleeding and vascular complications throughout the hemostasis period.

### Assessment of RAO

Radial artery patency was assessed by Doppler ultrasonography before discharge. RAO was defined as the absence of antegrade flow within the radial artery lumen. Patients were subsequently classified into two groups according to the presence or absence of RAO.

### Laboratory analyses and calculation of SCII

All laboratory analyses were performed on peripheral venous blood samples obtained before the procedure. WBC, neutrophil, lymphocyte, and platelet counts were measured using a complete blood count analyzer (Sysmex XN-9000, Kobe, Japan), whereas C-reactive protein (CRP), fibrinogen, creatinine, glucose, albumin, and lipid parameters were measured using standard biochemical methods (Atellica system, Siemens, Germany).

The SCII was calculated using the following formula: SCII = (platelet count × fibrinogen)/(WBC count × 100). Platelet and WBC counts were expressed as × 10^3^/µL and fibrinogen as mg/dL. This formula is equivalent to the previously described calculation (platelet × fibrinogen (g/L)/WBC); because fibrinogen was measured in mg/dL, division by 100 provides the conversion to g/L [10]. Additionally, other inflammatory indices were calculated using the following formulas: Systemic Immune–Inflammation Index (SII) = platelet × neutrophil/lymphocyte counts, NLR = neutrophil/lymphocyte counts, PLR = platelet/lymphocyte counts [10].

### Statistical analysis

Statistical analyses were performed using IBM SPSS Statistics for Windows, version 27.0 (IBM Corp., Armonk, NY, USA). The distribution of continuous variables was assessed with the Kolmogorov–Smirnov test. Normally distributed continuous variables were expressed as mean ± standard deviation, non-normally distributed variables as median (interquartile range), and categorical variables as numbers and percentages. Between-group comparisons were made using the independent-samples *t*-test or the Mann–Whitney U test for continuous variables and the Chi-square test for categorical variables. To identify variables associated with RAO, univariate logistic regression analysis was performed first, and variables with P < 0.05 in the univariate analysis were entered into a multivariable logistic regression model. The components of SCII—platelet count, fibrinogen, and WBC—were not included in the same multivariable model as SCII in order to avoid multicollinearity, and collinearity within the model was evaluated using the variance inflation factor (VIF). Results were reported as odds ratios (ORs) with 95% confidence intervals (CIs). The discriminative performance of SCII, SII, NLR, and PLR for predicting RAO was evaluated by receiver operating characteristic (ROC) curve analysis; the area under the curve (AUC) was calculated, and the optimal cut-off value was determined according to the maximum Youden index, with the corresponding sensitivity and specificity reported. A two-sided P value < 0.05 was considered statistically significant.

## Results

A total of 2,055 patients who underwent elective transradial CAG or PCI were included in the study. RAO occurred in 96 patients (4.7%), whereas 1,959 patients (95.3%) did not develop RAO (Table 1).

**Table 1:** Baseline Clinical, Procedural, and Laboratory Characteristics of Patients According to RAO Status

Variables	RAO (+) (n = 96)	RAO (–) (n = 1,959)	P value
Age (years)	55.4 ± 11.4	60.5 ± 12.1	< 0.001
Male sex, n (%)	58 (60.4%)	1390 (71.0%)	0.033
Hypertension, n (%)	47 (49.0%)	920 (47.0%)	0.667
Diabetes mellitus, n (%)	11 (11.5%)	98 (5.0%)	0.008
COPD, n (%)	7 (7.3%)	119 (6.1%)	0.640
Stroke, n (%)	3 (3.1%)	39 (2.0%)	0.489
Atrial fibrillation, n (%)	5 (5.2%)	78 (4.0%)	0.796
Medications			
ASA, n (%)	41 (42.7%)	803 (41.0%)	0.753
P2Y12 inhibitors, n (%)	33 (34.4%)	685 (35.0%)	0.885
Oral anticoagulants, n (%)	6 (6.2%)	84 (4.3%)	0.408
Statins, n (%)	38 (39.6%)	627 (32.0%)	0.210
Procedure type			
PCI	19 (19.8%)	594 (30.3%)	0.024
Laboratory			
Hemoglobin (g/dL)	14.2 ± 1.6	14.5 ± 1.4	0.093
WBC (10^3^/µL)	8.30 ± 1.46	8.54 ± 1.83	0.129
Neutrophil (10^3^/µL)	5.9 ±1.0	5.7 ± 1.0	0.115
Lymphocyte (10^3^/µL)	1.61 ± 0.58	1.73 ± 0.54	0.084
Platelet (10^3^/µL)	293 ± 65	257 ± 79	0.001
AST (U/L)	30 ± 18	27 ± 16	0.218
CRP (mg/L)	12.1 ± 5.6	10.8 ± 7.4	0.020
Creatinine (mg/dL)	1.08 ± 0.32	0.97 ± 0.38	0.004
Glucose (mg/dL)	106 ± 32	112 ± 35	0.114
Albumin (g/L)	42.3 ± 4.9	41.3 ± 5.3	0.128
Fibrinogen (mg/dL)	342 ± 68	308 ± 65	0.001
Triglyceride (mg/dL)	184 ± 69	173 ± 61	0.092
LDL (mg/dL)	115 ± 45	121 ± 47	0.105
HDL (mg/dL)	38 ± 9	40 ± 10	0.209
NLR	3.66 ± 1.20	3.30 ± 1.10	0.004
PLR	182 ± 52	149 ± 46	< 0.001
SII	1070 ± 380	850 ± 330	< 0.001
SCII	120.7 ± 36.4	93.1 ± 41.5	< 0.001

RAO: radial artery occlusion; COPD: chronic obstructive pulmonary disease; ASA: acetylsalicylic acid; PCI: percutaneous coronary intervention; WBC: white blood cell count; AST: aspartate aminotransferase; CRP: C-reactive protein; LDL: low-density lipoprotein cholesterol; HDL: high-density lipoprotein cholesterol; NLR: neutrophil-to-lymphocyte ratio; PLR: platelet-to-lymphocyte ratio; SII: Systemic Immune–Inflammation Index; SCII: Systemic Coagulation–Inflammation Index.

The baseline clinical, laboratory, and procedural characteristics of the study population are presented in Table 1. Patients who developed RAO were significantly younger than those without RAO (55.4 ± 11.4 vs. 60.5 ± 12.1 years, P < 0.001). The proportion of male patients was lower in the RAO group (60.4% vs. 71.0%, P = 0.033), whereas diabetes mellitus was more prevalent among patients with RAO (11.5% vs. 5.0%, P = 0.008). PCI was performed less frequently in patients who developed RAO (19.8% vs. 30.3%, P = 0.024). No significant differences were observed regarding hypertension, chronic obstructive pulmonary disease, previous stroke, and atrial fibrillation between the groups. Baseline antithrombotic therapy, including aspirin, P2Y12 inhibitors, and oral anticoagulants, did not differ significantly between the groups (Table 1).

Regarding laboratory parameters, platelet count (293 ± 65 vs. 257 ± 79 × 10^3^/µL, P = 0.001), CRP (12.1 ± 5.6 vs. 10.8 ± 7.4 mg/L, P = 0.020), serum creatinine (1.08 ± 0.32 vs. 0.97 ± 0.38 mg/dL, P = 0.004), and fibrinogen (342 ± 68 vs. 308 ± 65 mg/dL, P = 0.001) were significantly higher in patients with RAO. In contrast, hemoglobin, WBC count, neutrophil count, lymphocyte count, glucose, albumin, triglycerides, low-density lipoprotein (LDL) cholesterol, high-density lipoprotein (HDL) cholesterol, and aspartate aminotransferase (AST) did not differ significantly between the groups. In addition, inflammatory indices, including NLR (3.66 ± 1.20 vs. 3.30 ± 1.10, P = 0.004), PLR (182 ± 52 vs. 149 ± 46, P < 0.001), and SII (1,070 ± 380 vs. 850 ± 330, P < 0.001), were significantly higher in patients who developed RAO. Similarly, SCII was markedly elevated in the RAO group compared with the non-RAO group (120.7 ± 36.4 vs. 93.1 ± 41.5, P < 0.001) (Table 1).

Univariate logistic regression analysis demonstrated that age (OR: 0.966, 95% CI: 0.950–0.982, P < 0.001), male sex (OR: 0.638, 95% CI: 0.420–0.967, P = 0.034), diabetes mellitus (OR: 2.354, 95% CI: 1.232–4.499, P = 0.010), PCI (OR: 0.576, 95% CI: 0.345–0.960, P = 0.030), CRP (OR: 1.019, 95% CI: 1.005–1.039, P = 0.003), creatinine (OR: 2.030, 95% CI: 1.302–3.183, P = 0.002), and SCII (per 10-unit increase; OR: 1.189, 95% CI: 1.140–1.239, P < 0.001) were associated with RAO (Table 2).

**Table 2:** Univariate and Multivariate Logistic Regression Analyses for Predictors of Radial Artery Occlusion

Variables	Univariate OR (95% CI)	P value	Multivariate OR (95% CI)	P value
Age, years	0.966 (0.950–0.982)	< 0.001	0.966 (0.949–0.983)	< 0.001
Sex	0.638 (0.420–0.967)	0.034	0.701 (0.453–1.083)	0.109
DM	2.354 (1.232–4.499)	0.010	2.342 (1.203–4.560)	0.012
PCI	0.576 (0.345–0.960)	0.030	0.614 (0.361–1.042)	0.068
CRP (mg/L)	1.019 (1.005–1.039)	0.003	1.015 (0.987–1.044)	0.285
Creatinine (mg/dL)	2.030 (1.302–3.183)	0.002	1.340 (0.801–2.252)	0.270
SCII (per 10-unit increase)	1.189 (1.140–1.239)	< 0.001	1.174 (1.120–1.228)	< 0.001

CI: confidence interval; OR: odds ratio; DM: diabetes mellitus; PCI: percutaneous coronary intervention; CRP: C-reactive protein; SCII: Systemic Coagulation–Inflammation Index.

Variables with P < 0.05 in the univariate analysis were entered into the multivariable logistic regression model. In multivariable analysis, age (OR: 0.966, 95% CI: 0.949–0.983, P < 0.001), diabetes mellitus (OR: 2.342, 95% CI: 1.203–4.560, P = 0.012), and SCII (per 10-unit increase; OR: 1.174, 95% CI: 1.120–1.228, P < 0.001) remained independent predictors of RAO, whereas sex (P = 0.109), PCI (P = 0.068), CRP (P = 0.285), and creatinine (P = 0.270) lost statistical significance (Table 2).

**Figure 1:**
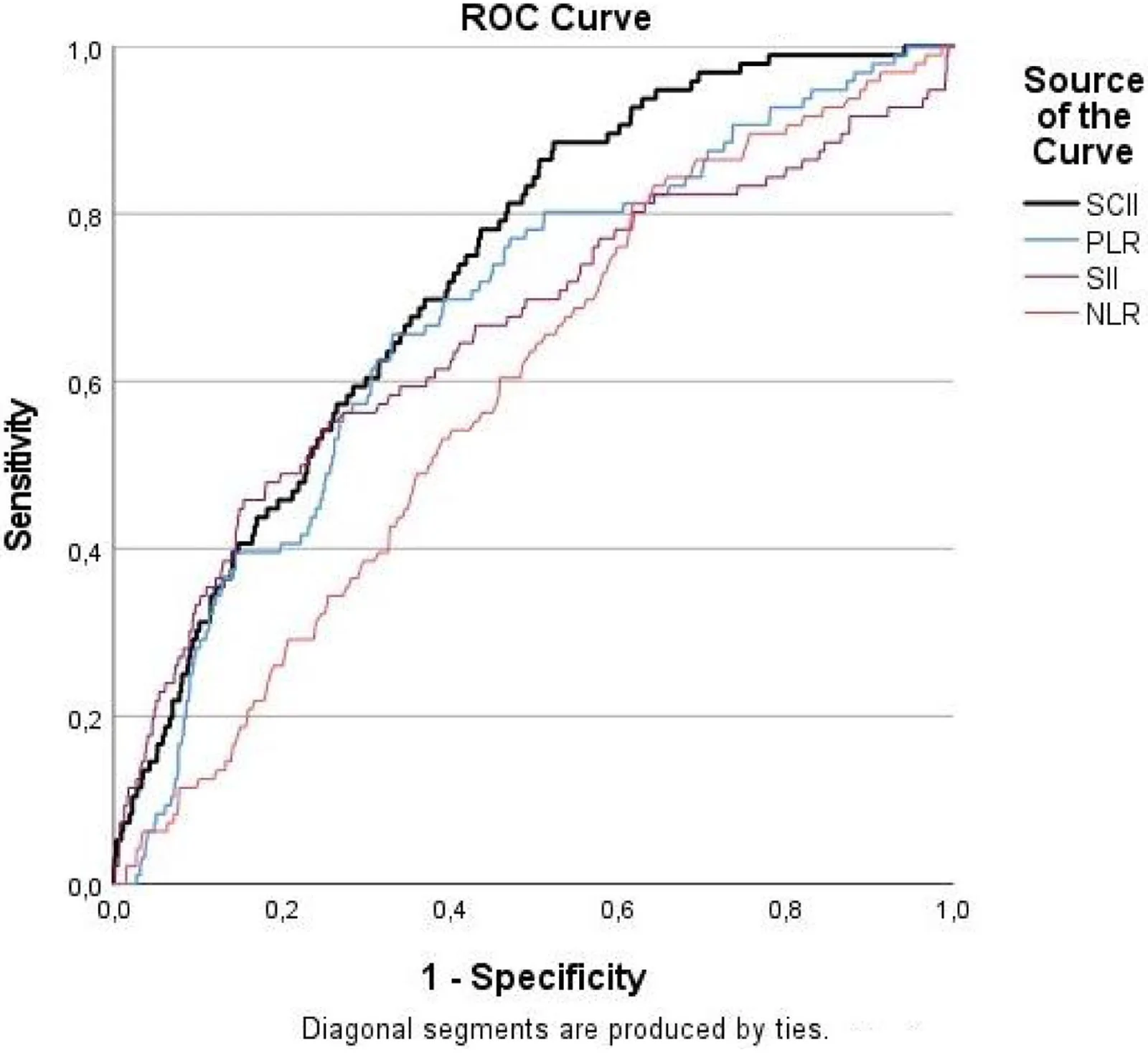
Receiver operating characteristic (ROC) curve of the SCII, PLR, SII, and NLR for predicting radial artery occlusion after transradial coronary angiography. SCII: Systemic Coagulation–Inflammation Index; PLR: platelet-to-lymphocyte ratio; SII: Systemic Immune–Inflammation Index; NLR: neutrophil-to-lymphocyte ratio.

ROC curve analysis demonstrated that SCII had a moderate discriminative ability for predicting RAO (AUC: 0.730, 95% CI: 0.686–0.774, P < 0.001) followed by PLR (AUC: 0.678, 95% CI: 0.625–0.732, P < 0.001), SII (AUC: 0.659, 95% CI: 0.594–0.723, P < 0.001), and NLR (AUC: 0.589, 95% CI: 0.536–0.643, P = 0.003) (Fig. 1; Table 3). The optimal cut-off value of SCII was 101.2, providing a sensitivity of 70.4% and a specificity of 57.7%.

**Table 3:** Receiver Operating Characteristic Analysis of SCII, PLR, SII, and NLR for Predicting Radial Artery Occlusion

Variable	AUC	95% CI	P value
SCII	0.730	0.686–0.774	< 0.001
PLR	0.678	0.625–0.732	< 0.001
SII	0.659	0.594–0.723	< 0.001
NLR	0.589	0.536–0.643	0.003

AUC: area under the curve; CI: confidence interval; SCII: Systemic Coagulation–Inflammation Index; PLR: platelet-to-lymphocyte ratio; SII: Systemic Immune–Inflammation Index; NLR: neutrophil-to-lymphocyte ratio.

## Discussion

The main findings of this study can be summarized as follows: (1) the incidence of RAO was 4.7% in patients undergoing elective transradial coronary procedures, and SCII levels were significantly higher in patients who developed RAO; (2) in multivariable analysis, age, diabetes mellitus, and SCII were identified as independent predictors of RAO; and (3) SCII showed a moderate discriminative ability for predicting the development of RAO (AUC = 0.730). Among the evaluated inflammatory indices (NLR, PLR, and SII), SCII achieved the highest predictive performance. These findings suggest that SCII, which jointly reflects inflammation and coagulation activation, may be a useful biomarker for predicting RAO after transradial procedures.

With a reported incidence of 1% to 10%, RAO remains the most common vascular complication of transradial procedures [3]. The 4.7% incidence observed in our study is consistent with the rates reported in contemporary series using patent hemostasis; for example, the proRadial study reported an incidence of 4.6% [13]. Because inflammation and thrombosis play central roles in its pathogenesis, it is expected that biomarkers jointly reflecting these processes would be associated with RAO.

In our study, diabetes mellitus was identified as an independent predictor of RAO, a finding consistent with the existing literature [14, 15]. In diabetes, the prothrombotic milieu created by endothelial dysfunction, platelet hyperreactivity, oxidative stress, and chronic low-grade inflammation facilitates thrombus formation following procedure-related vascular injury [16]. In addition, the lower proportion of male patients in the RAO group reflects a well-established association, explained by the smaller radial artery diameter and consequently higher sheath-to-artery ratio in women [14, 17, 18]. In contrast, the younger age of patients who developed RAO is at odds with some studies reporting advanced age as a risk factor [19]; however, the relationship between age and RAO has not shown a consistent direction in the literature [4]. Indeed, some studies have also reported that patients who developed RAO were younger, a finding attributed to the smaller radial artery diameter in younger individuals [18, 20]. This finding may be related to population characteristics, radial artery anatomy, and the interaction between age and sex; the persistence of age as an independent predictor in multivariable analysis indicates that this relationship warrants further investigation.

In our study, the performance of PCI, CRP, and creatinine levels were associated with RAO in univariate analysis but lost their significance in multivariable analysis. The lower frequency of RAO in patients undergoing PCI may be explained by the higher procedural anticoagulation administered in this group; indeed, in our protocol, PCI patients receive a higher weight-adjusted heparin dose (100 IU/kg versus 5,000 IU for diagnostic procedures), and adequate intraprocedural anticoagulation has been shown to reduce RAO in previous studies [4]. Consistent with this finding, a contemporary cohort also reported a lower RAO incidence in PCI patients compared with diagnostic angiography (6.2% vs. 10.6%) [21]. Furthermore, the univariate association of CRP is consistent with the link between inflammation and radial artery thrombosis [22], whereas the association of creatinine may be explained by the endothelial dysfunction, chronic inflammation, and prothrombotic background accompanying impaired renal function [23]. Although the loss of statistical significance of CRP in the multivariable model may suggest that coagulation-related mechanisms play a more prominent role than inflammation alone in the development of RAO, our findings primarily support the importance of the interaction between inflammation and coagulation rather than either pathway alone. In this regard, the independent association of SCII suggests that a composite index integrating both inflammatory and coagulation pathways provides stronger prognostic information for the development of RAO.

SCII is a relatively novel biomarker that combines platelet count, fibrinogen level, and WBC count into a single formula, reflecting the interaction between the inflammatory and coagulation systems [8]. Fibrinogen is both one of the fundamental components of thrombus formation and a strong acute-phase reactant; platelets play a central role in thrombus formation, while leukocytes reflect the inflammatory response [24]. Given the bidirectional interaction between inflammation and coagulation, a high SCII value represents a biological state in which increased thrombotic tendency and systemic inflammation coexist. Because RAO develops through local inflammation and thrombus formation following endothelial injury, the independent association of high SCII levels with RAO is pathophysiologically consistent [3].

In recent years, the prognostic value of SCII in various cardiovascular and thrombotic conditions has been increasingly investigated; it has been reported to predict mortality in acute type A aortic dissection, coronary stent restenosis, bleeding risk in acute coronary syndrome, the development of septic embolism in infective endocarditis, and hemodialysis vascular access dysfunction [7–11]. Although the prognostic direction of SCII varies according to the clinical context, high SCII is associated with adverse outcomes in overtly thrombotic endpoints such as septic embolism [8]; the independent association of high SCII with RAO, a thrombotic complication, is likewise consistent with this framework. To the best of our knowledge, this is the first study to evaluate the relationship between SCII and RAO, and it provides an original contribution to the literature.

Clinically, SCII is a low-cost and widely accessible biomarker that can be readily calculated from a routine complete blood count and fibrinogen. Importantly, SCII remained an independent predictor of RAO after multivariable adjustment, suggesting incremental value beyond conventional risk factors. Given the low prevalence of RAO, SCII may have limited positive predictive value when used alone; however, identifying patients with high pre-procedural SCII values may be useful—as an additional layer to existing clinical and procedural risk factors—for prioritizing preventive strategies such as ensuring adequate anticoagulation, meticulously maintaining patent hemostasis, and more closely monitoring radial artery patency.

### Limitations

This study has several limitations. First, its retrospective, single-center design precludes any causal inference and may introduce selection bias and residual confounding despite multivariable adjustment. Second, SCII was measured only in the pre-procedural period, so its dynamic changes over time could not be evaluated. Third, some procedural variables that may influence the development of RAO, such as radial artery diameter and sheath-to-artery ratio, could not be analyzed. Finally, because the study population consisted only of patients undergoing elective procedures, the generalizability of the findings to other patient groups, such as those with acute coronary syndrome, may be limited.

### Conclusion

In conclusion, SCII is an independent predictor of RAO in patients undergoing transradial coronary procedures and shows a moderate discriminative ability. This index, which can be readily obtained from routine laboratory parameters, may be a useful tool for the early identification of high-risk patients and the planning of preventive strategies. Multicenter, prospective studies are needed to confirm our findings.

## Data Availability

The data supporting the findings of this study are available from the corresponding author upon reasonable request.

## Abbreviations

AST: aspartate aminotransferase
AUC: area under the curve
CAG: coronary angiography
CI: confidence interval
CRP: C-reactive protein
HDL: high-density lipoprotein
LDL: low-density lipoprotein
NLR: neutrophil-to-lymphocyte ratio
OR: odds ratio
PCI: percutaneous coronary intervention
PLR: platelet-to-lymphocyte ratio
RAO: radial artery occlusion
ROC: receiver operating characteristic
SCII: Systemic Coagulation–Inflammation Index
SD: standard deviation
SII: Systemic Immune–Inflammation Index
UFH: unfractionated heparin
VIF: variance inflation factor
WBC: white blood cell
